# Recent Advances in In Silico Target Fishing

**DOI:** 10.3390/molecules26175124

**Published:** 2021-08-24

**Authors:** Salvatore Galati, Miriana Di Stefano, Elisa Martinelli, Giulio Poli, Tiziano Tuccinardi

**Affiliations:** 1 Department of Pharmacy, University of Pisa, 56126 Pisa, Italy; salvatore.galati@phd.unipi.it (S.G.); miriana.distefano@phd.unipi.it (M.D.S.); e.martinelli3@studenti.unipi.it (E.M.); tiziano.tuccinardi@unipi.it (T.T.); 2 Center for Biotechnology, Sbarro Institute for Cancer Research and Molecular Medicine, College of Science and Technology, Temple University, Philadelphia, PA 19122, USA

**Keywords:** target fishing, reverse screening, molecular similarity, machine learning, docking

## Abstract

In silico target fishing, whose aim is to identify possible protein targets for a query molecule, is an emerging approach used in drug discovery due its wide variety of applications. This strategy allows the clarification of mechanism of action and biological activities of compounds whose target is still unknown. Moreover, target fishing can be employed for the identification of off targets of drug candidates, thus recognizing and preventing their possible adverse effects. For these reasons, target fishing has increasingly become a key approach for polypharmacology, drug repurposing, and the identification of new drug targets. While experimental target fishing can be lengthy and difficult to implement, due to the plethora of interactions that may occur for a single small-molecule with different protein targets, an in silico approach can be quicker, less expensive, more efficient for specific protein structures, and thus easier to employ. Moreover, the possibility to use it in combination with docking and virtual screening studies, as well as the increasing number of web-based tools that have been recently developed, make target fishing a more appealing method for drug discovery. It is especially worth underlining the increasing implementation of machine learning in this field, both as a main target fishing approach and as a further development of already applied strategies. This review reports on the main in silico target fishing strategies, belonging to both ligand-based and receptor-based approaches, developed and applied in the last years, with a particular attention to the different web tools freely accessible by the scientific community for performing target fishing studies.

## 1. Introduction

The identification of potential targets for a known bioactive compound is fundamental for drug design and development. Over the past few decades, a considerable number of chemical compounds have failed to obtain approval and reach the market due to severe clinical side effects and cross-reactivity that are observed during later stage clinical trials. “One-target, one-drug, one-disease” has been the dominant concept in traditional drug discovery, but this paradigm implies that a drug is developed to modulate a single target for a specific disease, when it is well known that this does not often happen. A drug that has secondary targets may lead to undesirable side effects, but it may also provide more opportunities for identifying new therapeutic uses, leading to the so-called drug repurposing. Conversely, the relevance of polypharmacology is increased by the observation of complex biological systems and their correlation with complex diseases [1]. The concept of polypharmacology argues that a multi-target approach based on a network of drug-target interactions can outperform treatments based on a single target-activity [2]. On this basis, the need for multi-target drug development and discovery is a key challenge for the future of medicinal chemistry.

**Figure 1:**
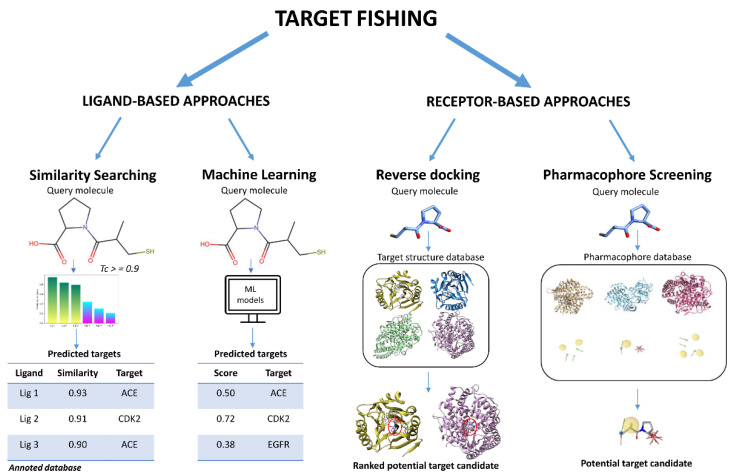
Overview of the main target fishing approaches.

Conventional methods for identifying potential targets with good accuracy include protein affinity isolation and subsequent mass spectrometric analysis, as well as approaches based on mRNA expression [3]. However, experimental approaches are expensive in terms of resources and time. Because of these limitations, in silico target fishing is considered a promising alternative for target identification. In contrast to virtual screening, which is used to search large libraries of compounds for molecules that are most likely to bind a specific target, the aim of target fishing, also known as in silico reverse screening, is to identify the most likely targets of a query molecule. Considering this, this approach may be more properly defined as in silico target-to-ligand matching; however, the term in silico target fishing is the most widespread and used in the drug discovery field, and will thus, herein, be employed for referring to this method. This approach allows not only the prediction of a potential drug-target interaction and thus the mechanism of action of the bioactive molecule, but also the prediction of adverse effects [4] and the evaluation of possible polypharmacology [5,6] and drug repurposing [7] applications. The computational strategies employed in target fishing can be classified into two categories based on the type of data used: ligand-based and receptor-based methods (Figure 1). In many cases, most of these methods have been implemented into computational tools or web servers for the convenience of researchers and they are often freely available. The ligand-based methods are more advantageous in large-scale virtual screening than structure-based methods because of the lower computational requirements, higher flexibility, and for the greater possibility of using machine learning. The ligand-based approaches are clearly preferable when a molecule shows a reasonable similarity to already known compounds. By contrast, receptor-based strategies such as docking-based approaches have the advantage that they can be used to predict molecules that represent previously unexplored chemical space. Furthermore, the ligand-based approach allows its application in the absence of structural knowledge of the target receptors, unlike the receptor-based methods that require structural information related to potential receptor targets. This is the case of reverse docking, which consists in evaluating the possible binding mode of a query molecule within the binding site of multiple protein targets in order to identify proteins with strong binding affinity for the query ligand. However, with the development of proteomics, the creation of new free-to-use, updated, and comprehensive protein databases, as well as the availability of a higher number of crystal structures, the use of receptor-based methods have also been furthered investigated, especially within the pharmacophore approach. Moreover, the ligand-based strategies are often combined with the receptor-based strategies with the aim of identifying new targets and biological activities for query molecules. For this reason, this review aims to outline the basic principles of these two types of target fishing approaches, focusing on methods and applications reported from 2015 to today, with a particular attention to the different web tools freely accessible by the scientific community for performing target fishing studies.

## 2. Ligand-Based Approaches

Ligand-based approaches are standard target fishing strategies widely used due to their independence from protein structure availability and the simplicity of their application. Therefore, non-expert users are able to employ these methods, usually providing reliable results in activity predictions and SAR analysis [8]. The logic of this strategy is based on the principle of similarity, according to which two compounds that have similar structural patterns can share similar bioactivities, thus interacting with similar targets. For this reason, ligand databases with activity annotation on known targets are necessary in order to perform a ligand-based reverse screening. The increased availability of chemical data in recent years has made it easier to obtain information about target-compound interactions reported on publicly available databases, such as ChEMBL [9] and PubChem [10], and about FDA-approved drugs or drug candidates, such as those included in the freely accessible DrugBank [11] and Therapeutic Target Database (TTD) [12,13].

Structural similarity assessment can be performed in two main ways: using either 2D or 3D approaches. The 2D strategy is based exclusively on the identification of shared chemical substructures between two compounds. In order to apply this method, two main components are necessary. The first is a molecular representation that can encode the chemical features of the compounds, while the second essential component is a similarity function that returns a numerical value or coefficient indicating the similarity between two compounds based on their molecular representation. Molecular representation often relies on universal descriptors that capture graph information such as fragments or topological environments of atoms. This method, known as 2D Fingerprint, employs a vector of bits to represent the structural properties [14]. The similarity coefficient measures the similarity of two bit vectors considering either shared or unshared chemical features. There are several similarity coefficients such as Euclidean, Tversky, and Dice, although the most widely used is the Tanimoto index (Ti), which consists of the ratio of the number of features shared by two molecules to the total number of features [15]. Conversely, the basis of the 3D strategy is that two molecules with similar volumes, occupying similar portions of space, may have similar biological properties [16]. This theory is supported because compounds are inherently three-dimensional, and their molecular conformations generally have a higher information content than their corresponding molecular graphs. The 3D structural similarity occasionally considers additional properties such as pharmacophores [17] or electrostatic information [18,19]. One of the major problems of the 3D ligand-based strategy is the lack of availability of bioactive 3D conformations and this limitation often influences the choice of researchers in developing new ligand-based approaches for target fishing purposes. For both ligand-based strategies, the potential targets of the query molecules are identified by looking at the annotated targets of the most similar compounds. In addition, certain methods assign a score to the potential predicted targets in order to create a ranking of the most likely targets, while reducing the probability of false positives. In the following sections, an overview of recent ligand-based strategies for target fishing are discussed, with particular attention to the web tools currently available (Table 1).

### 2.1. 2D-Structure Similarity Searching

The most straightforward approaches are based on the correlation between structural and biological properties of small molecules. These approaches rely on the simple analysis of known bioactive compounds, with annotated target-related activity information that are most similar to the query molecule. This type of approach strongly depends on the features used to compare molecular structures; therefore, the choice of descriptors, as well as the similarity index used, have significant influence. One of the points of debate of this methodology lies in the number of compounds and their annotated targets to be considered for prediction purposes. The classical strategy involves ranking the matched molecules according to their similarity index with respect to the query compound and choosing the top-N molecules for the final prediction. The choice of the N value naturally influences the number of targets predicted, as well as the presence of false positives. One of the latest reliable 2D ligand-based platforms for target fishing is MolTarPred web tool, [20] based on a pure 2D similarity search that employs the widely used ECFP4 fingerprints [21]. Peón and co-workers showed that considering the 10 most similar molecules to the query compound was a good compromise between successfully predicting a sufficient number of known targets for the query and limiting the number of false positives. The choice of this threshold was based on a study performed by the same authors: when fewer than 10 top-hits were considered, the method was likely to predict fewer true targets for the query molecule. Conversely, when more than 10 top-hits were selected, the predictive accuracy was generally worse. According to a reliability score based on matched molecules, a predicted target with a score of 10 showed to be a true target in 93% of cases [22].

The issue of false positives was investigated by Wang and collaborators, who developed the TargetHunter platform that performs a similarity search based on ECFP6 fingerprints and Ti score [23]. Their analysis revealed that matched molecules with a similarity index in the range of 0.3–0.4 possessed a low probability of reporting true annotated targets for the molecules used as queries. These results showed that a threshold of at least 0.5 is recommended to reduce the number of false positive targets.

A different approach used to rank the potential targets of a query molecule identified through a ligand-based similarity search was effectively applied in the MolTar platform developed by Liu and collaborators, which calculates, for each target, the average similarity of the K compounds most similar to the query (K-Nearest Neighbor approach) [24]. A previous work of the same research group investigated which is the best number of compounds (K) to consider for calculating the target score. Specifically, a 10-fold cross validation method was used to evaluate the influence of K on the performance of the target fishing approach. This validation strategy showed that an approach employing three nearest neighbor compounds (K = 3) outperforms a five nearest neighbor approach (K = 5) [25].

Alberga and co-workers recently developed the Multifingerprint Similarity Search Algorithm (MuSSeL) [26], a recent innovative search algorithm that uses several types of chemical fingerprints in combination. In this protocol, for each query-ligand pair, the Ti is computed using the 13 different fingerprints available in the python packages RDKit [27] and Pybel, [28] as well as in the CDK Java package [29]. By using this strategy, a potential target of a given query molecule is predicted as reliable if the corresponding ligand of the target shows sufficient structural similarity (in terms of Ti) to the query based on multiple fingerprint representations, in a sort of consensus fingerprint similarity approach. The strictness of the approach can thus be modified based on the Ti cut-off and the minimum number of fingerprints for which the ligand-query Ti value must satisfy the cut-off. An additional process provides a prediction of the biological activity of the query molecule by considering the average annotated activities of the matched compounds. The prediction of IC_50_ or K*_i_* is performed only if two conditions are satisfied: (i) there is a minimum number of fingerprints that gives the prediction, (ii) the difference between the maximum and minimum activity of the matched compounds is less than one logarithmic unit.

Although all methods described above appear reliable, the use of ligand datasets with different sizes can be a bias in the calculation of similarity to the query molecule. Particularly, in the case of small ligand datasets, a low baseline similarity may influence the choice of a given target favoring those with larger ligand datasets. To overcome this challenge, statistical methods have been proposed as a viable alternative for rescoring. In a general way, statistical methods process the significance of the interaction probability in order to provide a reliable and unbiased ranking based on a new scoring [30]. This type of statistical analysis is implemented in the PPB web platform, which employs the Manhattan similarity index and 10 different types of fingerprints, including fused fingerprints [31]. Target identification is performed by considering the similarity between the query and the most similar molecule of a group of compounds associated with the target. To identify the probability that the target-query association is random, the *p* value is calculated. The calculation is performed by generating similarity distances for each target present in the curated target dataset with each fingerprint. For each target, ChEMBL associated compounds and those randomly selected from the ZINC database are considered. Using a binomial distribution, the predicted targets for a given query molecule are ranked according to their *p* value, regardless of the size of the respective ligand set.

### 2.2. Mixed 2D/3D-Structure Similarity Searching

Although 2D strategies usually outperform 3D methods [32], scaffold diversity can be a problem with the 2D approach, as this method is generally limited to known active chemical scaffolds. In contrast, a 3D approach can overcome this problem and the results can be used to explore new scaffold models [33]. To overcome the limitations of 2D and 3D strategies, the SwissTargetPrediction platform combines 2D and 3D similarity and provides a target score derived from a cross-validation analysis [34]. This platform provides a web tool with a user friendly graphical interface that allows both non-experts and specialists to perform reverse screenings, employing a carefully prepared and ready-to-use chemical library. The dataset consists of 280,381 total small molecules interacting with 2686 targets, which were retrieved from the ChEMBL database, version 16, using only high confident data selected applying stringent criteria, thus including only compounds for which a value of K*_i_*, K_d_, IC_50,_ or EC_50_ lower than 10 μM for a corresponding target was reported. The quantification of similarity involves a 2D approach based on the Tanimoto index between path-based binary fingerprints (FP2) [35] and a 3D approach based on Manhattan distance similarity between Electroshape 5D (ES5D) vectors [36]. The SwissTargetPrediction model, which was trained by fitting a multiple logistic regression on various size-related subsets of known actives, returns a combined-score for each test compound, with respect to the query molecule. If the combined-score for a query-test compound pair is higher than 0.5, the query molecule is likely to share a protein target with the test compound. Another performing platform is chemical 3D Similarity Network Analysis Pulldown (CSNAP3D), developed by Lo and co-workers. The platform works using an interesting protocol, with a 3D similarity metric that considers both shape and pharmacophore scoring [17]. In this approach, eight 3D similarity metrics were evaluated based on molecular shape, pharmacophore, or a combination of shape and pharmacophore points. Metrics regarding 3D shape similarity were determined by the percentage of overlapped molecular features between two aligned molecules, while pharmacophore similarity metrics were defined by chemical matching of pharmacophore points. A new ligand alignment and scoring procedure called “ShapeAlign” was introduced. ShapeAlign performed an initial shape alignment between query and reference compounds, calculating a combination of shape Tanimoto index and the number of matching pharmacophore points. After calculating the similarity among query and compounds from the dataset, a network-based scoring function was used to predict the drug targets. Specifically, Lo and co-workers applied a consensus statistic (S-score) to identify the most common drug targets in the first-order neighbor of each query compound in the network. With a subsequent analysis, the authors used the CSNAP3D algorithm to identify several novel low molecular weight taxol mimetics. Thanks to an enrichment analysis on 206 benchmark compounds, the innovative ShapeAlign protocol introduced in this platform, in combination with 2D fingerprints, was allowed to outperform commonly used target prediction methods such as similarity ensemble approach (SEA) [37] and prediction of activity spectra for substances (PASS) [38].

### 2.3. Machine Learning

In recent years, the application of machine learning (ML), an essential component of artificial intelligence (AI), has become an attractive approach in computational chemistry, particularly in drug discovery [39], with significant advances [40]. The increased use of ML is linked to the increased generation of data derived from biological assays, whose analysis is increasingly challenging due to the large amount of information to be managed. The application of ML has accelerated the development of reliable and effective workflows to identify molecular patterns and predict biological properties of interest when a large amount of data is used [41]. ML techniques are divided into two main categories, namely supervised and unsupervised learning methods. In this analysis, we focused on supervised learning methods, which derive patterns (and thus learn) from training samples with known labels in order to determine the labels or classes of new samples. Among the ML algorithms for supervised learning that have been used in drug discovery, random forest (RF) [42], naive Bayesian (NB) [43] and support vector machine (SVM) [44] are the most widely used. Since target fishing aims to classify a compound as active against a spectrum of targets [45], ML classification models have proven to provide effective approaches for this purpose, resulting in the development of several protocols. Due to the necessity to perform multiple predictions in a typical target fishing protocol, the ML techniques used in this field can be classified into three broad categories, which are analyzed below: classical quantitative structure-activity relationships (QSAR), proteochemometrics-based (PCM) strategies, and model-stacking approaches [46].

The simplest and most common strategy based on the conventional QSAR approach, called multi-targets QSAR, involves the generation, or training, of different single-target models for each protein target considered within the target fishing approach [47,48,49]. Conversely, the opportunity to manipulate multiple predictions simultaneously makes the multi-classes strategy a promising application [50]. In simplest terms, the main difference between the two methodologies is the number of models used to achieve the target prediction. In a multi-targets approach, each model is trained to learn patterns that can discriminate active compounds from inactive compounds against single targets. Thus, the final prediction is obtained by collecting the results provided by each model (one per target). Conversely, in a multi-class strategy there is only one trained model that generates a vector for each query molecule, whose length corresponds to the number of targets used to train the model. Thus, using a single prediction (binary or regression vector) it is possible to obtain an overview of the putative biological spectra of the required compound. The main methods that allow the simultaneous prediction of activity on multiple targets are based on deep neural network (DNN) architectures [51]. Different studies have shown that multitask deep neural network (MT-DNN) modeling can increase the predictive performance compared to other ML methods [52], although the problem of missing data in multi-target matrices still represents a constantly studied topic [53]. This problem arises from the type of data used in a multi-class approach, consisting of a matrix in which each row corresponds to a single molecule associated with various target activities (stored in different columns): this is called binary classification or measured activity. Data sets used for multi-class prediction studies should be complete, which indicates that each compound should have been tested across the entire target set and should thus present activity values related to all targets considered within the matrix. However, this is not always possible and often leads to sparse arrays in which molecules miss the necessary activity values. To overcome this problem, in many applications, compounds are assumed as inactive against those targets for which the activity record is missing, which can lead to false negatives [54]. The conventional multi-targets approach has been used by Lee and co-workers to develop a QSAR-based web-implemented platform [55], which collects a different RF model for each of the 1121 targets included in their dataset. The various models were trained with data collected from ChEMBL database. Ligands were defined as actives toward a certain target if a related IC_50_, EC_50_, K*_i_*, or K_d_ value below 10 μM was available, while they were considered as inactive in absence of any type of activity reported. The efficiency of the approach was evaluated with an internal five-fold cross-validation and a further external validation. Using a strategy to transform the model score into interaction probabilities, the predicted targets were ranked. This approach showed good results; in particular, the recall rates obtained were 67.6% and 73.9% for the top 1% and 3% of targets, respectively.

In contrast to QSAR, PCM modeling is based on the use of descriptors, in order to represent a dataset of ligands, which also provide information about the corresponding protein or target. Therefore, a PCM model is designed considering both ligand and target characteristics [56]. In certain cases, the PCM approach demonstrated to outperform conventional QSAR strategies [57,58], although, in other studies, the two methods performed similarly [59]. An application of PCM in the target fishing field was realized by Wen and collaborators, which applied deep-learning deep-belief network (DBN) models to accurately predict new potential targets of FDA approved drugs [60]. The comparison with popular algorithms such as random forest, Bernoulli naive Bayesian, and decision tree showed that the DBN achieved the best performance with an accuracy value of 0.86. This evaluation indicates that DBN models together with PCM can be profitably used for the prediction of new targets. The combined use of QSAR and PCM models was also used by Paricharak and collaborators for developing an integrated drug discovery pipeline allowing to evaluate the polypharmacology of compounds and obtain insights into the potency and affinity of small molecules for specific targets [61]. The QSAR-based target prediction model, employing a Laplacian-modified naive Bayesian classifier, was trained using 53,084 ligand-target associations (for a total of 262,174 compounds), considering 3481 protein targets, while the PCM models were focused on a dihydrofolate reductase (DHFR) target, being trained on a dataset including 20 eukaryotic, protozoan, and bacterial DHFR sequences, and 1505 DHFR inhibitors, for a total of more than 3000 data points. The combined approach was then used to identify potential plasmodial DHFR inhibitors among the GlaxoSmithKline (GSK) Tres Cantos Antimalarial (TCAMS) [62], which contains more than 13,000 compounds that experimentally inhibit the growth of P. falciparum. By combining the findings obtained from the QSAR-based target prediction model and the PCM approach, 23 compounds were identified as potential high affinity DHFR inhibitors.

Recently, the advent of model stacking has become popular in chemoinformatics and therefore attractive to propose new strategies for target prediction [63]. The stacking technique is a two-level hierarchical framework that consists of training a model (meta-learner or second training level) using the predictions obtained from several other models (first training level) as features [64]. The purpose behind model stacking, similar to consensus strategies, is that the use of a combination of models can lead to an improvement in model predictions compared to using single models alone. One of the most recent implementations of stacking learning is the STarFish platform with its web application [65]. The challenge that led to the development of this platform was to investigate how a computational target fishing approach built with synthetic data can correctly and reliably predict new targets for natural products. The benchmark dataset used for model building consisted of 1943 unique compounds and 103 targets, for a total of 5589 target-ligand pairs. The comparison of random forest (RF), k-nearest neighbors (KNN) and multi-layer perceptron (MLP) models, either unstacked or stacked in different combinations, showed that the stacking approach performed better. In particular, the best performance combination was achieved with a training level 0 consisting of KNN and RF, followed by a training level 1 consisting of logistic regression. Although the above combination obtained the best performance, the platform was implemented to use a stacked model including only KNN in training level 0 due to the still high performance (similar to the best combination) obtained with a significantly lower computational expense.

## 3. Receptor-Based Approaches

Receptor-based methods consist of approaches that use protein structural information in order to predict ligand–target pairs. These methods predict not only the potential off-targets of small molecules, but also their putative binding modes within the receptors, which are necessary for understanding their mode of action and rationally designing selective compounds. These methods mainly include reverse docking and pharmacophore-based target fishing. The receptor-based approaches are dependent on three-dimensional (3D) protein structures. While receptor-based pharmacophore searches need at least one reference co-crystallized complex as input, docking-based target prediction needs only the 3D structure of the target and the active site location, which can be identified by a co-crystallized ligand or through pocket identification algorithms [66]. Thanks to the improvements in proteomics, it is easier to obtain information on protein structures and to find key residues within a binding pocket, thus detecting potential receptor-based pharmacophore features describing ligand moieties able to bind such key residues. In this sense, reverse docking is the most straightforward computational approach to predict whether a ligand may bind to a macromolecular target; the predicted ligand-protein binding interactions are then useful sources for lead optimization. However, there are issues associated with reverse docking, such as the generation of a suitable target datasets, inability to use receptor flexibility due to high computational cost, and the inaccurate prediction of binding free energy often employed for target ranking. In the following section, the latest example of reverse docking and pharmacophore-based target fishing strategies are reported, with a more comprehensive analysis of the available web tools (Table 2).

### 3.1. Reverse Docking

In contrast to traditional molecular docking, reverse docking, also known as inverse docking, is a powerful method for identifying potential protein targets for a given small-molecule ligand among a large number of protein targets. This approach has proved to be valid for different applications, from adverse side effect predictions to drug repositioning and lead optimization studies [67]. The main steps necessary to perform a reverse docking study are the generation of a target structure database, the prediction of energetically favorable binding conformations of the query ligand within the different targets, and their ranking according to their docking score. The first challenge to be faced is the recognition of the target binding sites, as it is not always possible to easily determine the active site of a protein, due to the unavailability of co-crystallized ligands. Therefore, an automatic procedure to achieve this task is desirable, considering the large number and variety of targets. For example, in the method described by Kunz and co-workers in 1982 [68] a group of overlapped probe spheres of specified radii was used to sample the protein surface and identify potential binding cavities. Another way to identify binding pockets relies on the use of specific site searching programs, such as fpocket [69] or SiteMap [70]. The construction of a suitable protein target database is a fundamental step for improving accuracy and reliability of reverse docking methods. These databases can be built by downloading a series of protein crystal structures from the Protein Data Bank (PDB), which should be properly processed prior to docking studies (e.g., by removing unnecessary water molecules and adding hydrogens). Protein flexibility represents another challenge to perform docking of ligands, since the binding site may exist in multiple shapes. Indeed, docking programs are not capable of performing molecular docking considering flexible proteins. The majority of docking algorithms treat the ligand as flexible by keeping the protein rigid and ignoring the ligand- and receptor-induced fit effects, while others perform docking on semi-flexible proteins, considering alternative conformations of specific residues side chains [71,72].

Finally, the last important step of reverse docking is the generation of a score in order to rank the potential targets of a query molecule. Generally, the binding energy calculated by the scoring functions of the docking software is used for ranking targets: a low docking energy should correspond to a stronger binding between ligand and protein. Several tools such as idTarget, [73] TarFisDock [74] and DPDR-CPI [75,76] base target ranking and prediction mainly on scoring functions. For example, idTarget employs a semi-empirical free energy function that includes weighting coefficients of hydrogen bonding, electrostatics, desolvation, and torsional entropy terms, whereas TarFisDock uses molecular mechanics energy functions only based on van der Waals and electrostatic terms. However, scoring functions are most often inaccurate for properly evaluating the ligand-protein binding affinity [77] and represent a limit for target ranking. For instance, the variance in size, shape, and solvent exposure of proteins binding sites generally leads to unbalanced scores across targets, presenting different binding pockets. In this regard, an interesting extensive performance assessment of docking-based target fishing approaches was conducted by Lapillo and co-workers [78]. By applying a consensus docking strategy combining the results of multiple docking procedures, they observed that the results of the target prediction were strongly related to the volume and shape of the target binding site. As the volume of the binding sites increased and the binding pockets became more open and solvent-accessible, the consensus among the different docking methods and the overall reliability of target prediction decreased, highlighting the close connection between these properties and the target prediction ability of the docking procedures. Other research groups developed different methods to normalize the docking scores, considering various features in binding cavities or combining docking scores with other topological evaluations such as interaction fingerprints. For instance, Wang and collaborators suggested a correction term to improve the target prediction performance based on the ratio between the hydrophobic and the hydrophilic surface areas in the binding site of the target protein [79]. Moreover, Liu and co-workers [80] showed that the use of protein−ligand interaction fingerprints (PLIFs) facilitated re-ranking of ligand docking poses based on their similarity to the known binding modes of relevant reference molecules. Other studies have revealed that ML models based on PLIFs outperformed docking scores for in silico screening [81]. In this context, Nogueira and Koch [82] proposed a docking-based target prediction approach by combining the protein atom score contributions-derived interaction fingerprints (PADIFs) and ML methods (either ANN or SVM). The authors tested their scoring functions on three validation data sets, and they observed that improved prediction performances with respect to classic scoring functions were obtained. Ultimately, probability scores were predicted and successfully used to rank the targets of experimentally active multi-target compounds for which activity data related to up to different targets per ligand were available.

The molecular docking programs used in reverse docking strategies work in a similar manner to conventional docking methods. The most important docking programs, such as AutoDock [83], DOCK [84] or Glide [85] have been used with a few modifications in reverse docking tools. We herein report examples of available target fishing tools based on reverse docking and their recent applications. INVDOCK [86], developed in 2001, is one of the first online services for ligand-protein reverse docking. INVDOCK has an in-house protein target database of 9000 protein and nucleic acid entries, and recognizes cavities on the protein surface as active binding pockets if these are covered by a highly concentrated cluster of spherical probes [68]. The simplified DOCK scoring function is used to estimate the ligand-protein binding energy. INVDOCK has been used for reverse docking in many studies [87,88,89,90,91,92] and it has recently been employed to analyze potential protein targets of polyphenols, as well as to evaluate their effects on Alzheimer’s disease [93].

TarFisDock [74] is a web-based tool, developed in 2006, for automating the procedure of searching for small molecule–protein interactions using a large set of protein structures. It includes the Potential Drug Target Database (PDTD), a database containing more than 1200 protein structures covering more than 800 known or potential drug targets [94]. The active site of each protein is defined by all residues within a 6.5 Å shell from the bound ligand. This tool also adopts DOCK software to calculate the binding energy between ligands and targets. Recently, TarFisDock was employed in a hybrid protocol that involved the combination of pharmacophore screening and docking to identify potential targets for 2-thiazolylimino-5-benzylidene-thiazolidin-4-one scaffold, whose derivatives have shown antibacterial activity in in vitro tests comparable to drugs on the market [95].

idTarget [73] is another web server for reverse docking. Different from other tools and reverse docking servers, idTarget performs screenings that employ all protein structures deposited in the PDB, using the “divide-and-conquer” approach to find potential binding sites. MEDock and AutoDock4 are used as a docking engine and scoring function, respectively. Recently, Liu and co-workers predicted the drug targets of amino alcohols for studying the mechanisms of active compounds against Echinococcus species, responsible for an important parasitic disease that threatens human health and animal husbandry worldwide [96]. In this work, the 11 most active amino alcohols were submitted to the idTarget server, and the inverse docking result list presented only the top 200 proteins based on their binding energy. Then, according to the frequency and binding energy, they manually selected the most common targets. Corresponding three-dimensional structures of the potential drug targets were built after sequence analysis and homology modeling. After further screening by molecular docking, the activities of the candidate targets were validated in vitro, identifying glycogen phosphorylase as a potential drug target for amino alcohols.

A docking-based web server that employs a different strategy is the DPDR-CPI [76] server, which corresponds to an upgraded version of DRAR-CPI [75] and is used to perform drug repositioning via chemical-protein interactome (CPI). This method uses an interaction strength matrix of drugs across multiple human proteins, and it aims at exploring unexpected drug-protein interactions. When the query molecule file is submitted, the compound is docked by AutoDock Vina against 611 targets with default parameters. The top-scored docking poses and their corresponding scoring values are extracted and sent to ML models for the generation of predictions. Luo and collaborators, which developed and evaluated DPDR-CPI, demonstrated the reliability of the tool using rosiglitazone, an anti-diabetic drug that has been on the market for years, as a test query molecule: the server successfully identified the original indications of rosiglitazione, i.e., hypoglycemia and diabetes mellitus, with high confidence. Moreover, Alzheimer’s disease, retinal disorders, and glaucoma were also prioritized among the top predictions, in agreement with the literature reports [76].

ACID [97] is a web platform with a user-friendly interface designed for drug repurposing to significantly reduce user time for data gathering and to allow a multi-step analysis without human supervision. It consists of the following three tools: (1) an automated consensus inverse docking protocol combining AutoDock Vina, LEDOCK, PLANTS, and PSOVina; (2) a compound database containing 2086 approved drugs with original therapeutic information; and (3) a known target database containing 831 protein structures from PDB, covering 30 therapeutic areas. The rationale behind the choice of a consensus inverse docking approach relied in the consideration that different docking methods use different conformational search algorithms and scoring functions; therefore, combining different docking software may be beneficial in terms of docking reliability and target identification, as it was proved to be in terms of pose prediction and hit finding [98,99]. To evaluate the performance of the ACID web server, Citalopram and Amitriptyline were used as query structures to find their target proteins and, in both cases, the tool successfully predicted the targets present in the literature.

Among the different reverse docking platforms, there are also examples of tools focused on predictions relative to specific protein families. Among these, DIA-DB [100] is a web server for the identification of potential antidiabetic drugs, which uses two different approaches relying on ligand-similarity and receptor-based virtual screening. DIA-DB employs inverse virtual screening of compounds with Autodock Vina against a given set of protein targets known to play a role in diabetes. A docking score for the query compound against the different targets is returned, as well as the structure of the predicted complexes and various graphical representations of the binding pockets. In a recent in silico study [101], a total of 867 compounds identified from African medicinal plants have been evaluated for their potential anti-diabetic activity and six of them have been identified as novel potential multi-targeted anti-diabetic compounds, with favorable ADMET properties for further drug development. Another tool belonging to this category is GUT-DOCK [102], a web service for docking of drug-like small molecules into G protein-coupled receptors. Most receptors included in GUT-DOCK belong to gut hormone receptors and other class B receptors, which are known to be expressed in the gastrointestinal tract. GUT-DOCK incorporates Autodock Vina [103] and OpenBabel [35] for ligand preparation and docking. GUT-DOCK provides immediate comparison of theoretical binding affinities calculated with docking and corresponding precomputed results obtained for betablockers of known diabetogenic effect, which is especially useful when the user’s compound is a beta-blocker.

### 3.2. Pharmacophore-Based Target Fishing Approach

The pharmacophore approach has been extensively used both in synthetic and computational chemistry. In computer-aided drug design, the pharmacophore-based approach is generally used for virtual screening in order to find those molecules presenting the structural moieties necessary for interacting with the desired target [104]. The pharmacophore approach in computational chemistry is appreciated for its efficiency and pragmatism, not only for identifying bioactive molecules [105], but also for recognizing and representing the key intermolecular interactions between proteins and ligands [106]. In a similar way, a single molecule with known pharmacophoric patterns can be used to identify potential target proteins and thus be used in target fishing strategies. The fundamental principle of receptor-based pharmacophore screening is that the binding of a compound to its protein targets is due to the presence of specific functional elements in the molecule that allow the formation of key ligand-protein interactions, which are represented by the pharmacophore [107]. For this reason, in order to build a pharmacophoric pattern, it is necessary to identify the different molecular features that are involved in ligand-protein interactions, which are generally labeled based on the type of interaction represented, such as H-bond donor or acceptors, aromatic, and hydrophobic features [108]. In this way, it is possible to create a database of receptor-based pharmacophore models related to many different target proteins; a specific query compound can be then screened against all pharmacophores of the database in order to find the best matches. The protein associated with the best matching pharmacophores should be the most likely to be a target of the query compound [109]. Usually, the features of a pharmacophore model are organized in a 3D arrangement and the pharmacophore can be either derived by direct identification of ligand-target interactions from experimental X-ray complexes or by recognition of common structural features identified from the superimposition of multiple reference active compounds sharing similar chemical moieties [110]. When applied to target fishing, a third method can be implemented, consisting of identifying the pattern of pharmacophoric requirements of the target protein, namely, the pattern of structural moieties or features a ligand should be endowed with, in order to properly interact with the target. However, the scarcity of information on protein conformation and the reduced availability of crystal structures made this last method more difficult to be employed until recent years. Currently, with the improvements in proteomics, it is easier to have information on protein structures before their biological activity is discovered, and thus find key residues within a binding pocket that can be used to obtain a potential pattern of required ligand pharmacophore features for future screening [111]. Another method for pharmacophoric residues identification consists of using chemical probes, usually water or organic solvents, and simulating their dynamic behavior on flexible molecular surfaces. This will allow identification of possible favorable interactions on the protein surface and convert them into pharmacophoric features [112]. A more recent method consists in identifying the energetic properties of the binding site and translate them into pharmacophoric patterns through the use of a common nearest neighbors (CNN) clustering method [113]. Moreover, through the use of 3D pharmacophore target fishing approaches, it is possible not only to identify target proteins, but to also predict the biological activity of the compounds screened against protein libraries [114].

With the increasing knowledge of protein structures and their physiological activity, new web services for pharmacophore-based target fishing and drug repurposing, as well as related online databases, were created. One of the first used web servers for drug repurposing is Drug REPOSitioning Exploration Source (Drug ReposER) [115]. Drug ReposER is able to facilitate the search and comparison among amino acid side chains that are similar to the ones found in binding sites of known drugs. This allows identification of new possible targets of known bioactive compounds according to three different options. The first takes into consideration all existing PDB structures searching for 3D patterns of residues that are similar to known drug-protein biding interfaces, while the second method searches only the query PDB structures provided by the user. The third method uses the known binding patterns of a molecule to discover possible interactions on new protein surfaces studying the molecule-protein complexes. Using this web server as a starting point, LigAdvisor [116] was created by Pinzi and co-workers to enable de novo drug discovery by ligand design and optimization and drug repurposing as well as polypharmacology studies. This platform is based on the similarity of 2D ligand-receptor interactions obtained from molecules belonging to the DrugBank and proteins contained in the PDB database. Moreover, it also contains information related to clinical trials and data reported in the Uniprot [117] database, which contains an average of 120 million entries belonging to human, animal, bacterial and viral proteomes, for a total of 84 thousand species. When employed for target fishing purposes, the user can also submit a query by drawing the chemical structures and the software reports of all the possible biological targets according to the binding interaction patterns available. Pinzi and collaborators tested the newly built LigAdvisor in applications of different case studies. As an example, the single-query search approach was used to predict the target of 22 potential repurposing candidates that were under clinical investigation for the treatment of Alzheimer’s disease [118]. According to the LigAdvisor predictions, 8 of the 22 investigated drugs were associated with at least one target related to Alzheimer’s disease.

Another recently developed tool, Protein-Ligand Interaction Profiler (PLIP) [119], is able to facilitate target identification and prediction through binding site alignment or similarity among molecular structures, residue sequences and ligand-protein interaction patterns. This web service analyzes non-covalent interactions within protein-ligand complexes belonging to PDB structures. The output information lists all the hydrogen and halogen bonds, water-bridged and salt-bridge interactions, and hydrophobic interactions as well as metal complexes, all detailed at atomic level. A recent application of PLIP involves the study of ligands and proteins with nucleic acids, allowing expansion of the scope of possible targets for new cancer drugs [120]. Another widespread tool is PharmMapper, a freely accessible web server for target identification based on the pharmacophore mapping procedure [121]. This tool can quickly identify potential target candidates due to its robust mapping method and its large in-house pharmacophore database, composed of over one-thousand models. The tool automatically finds the best poses for the query molecule screened against all the pharmacophoric models present in the protein databases, such as TTD and PDTD [94], and selects the best hits. PharmMapper has been used for identifying a wide variety of targets including those of capsaicin [122], salvianolic acid [123], and xanthorrhizol [124]. Moreover, this tool has resulted to be efficient in identifying the polypharmacological profile of drugs [30], allowing finding of more than one target for the same molecule, and contributing to the previously mentioned drug repurposing.

Another advantage of the pharmacophore-based target fishing is that it can be applied together with other in silico methods, such as reverse docking and QSAR studies [125]. The pharmacophoric approach can also benefit from the application of ML methods to efficiently identify new targets and biological activities of query compounds. In this context, SVM classifier-based models were used to predict if the query molecule is active or inactive towards the target and it was applied to natural compounds with biological activities, but unknown targets, by Rocha and collaborators [126]. This was achieved after having analyzed the 3D pharmacophore features for the molecule in all its interactions with the selected proteins in order to obtain a multi-conformational pharmacophore pattern. The latter was then assessed by the SVM model to check if there was an interaction with the different targets or not, also considering in vitro experimental data for the analyzed molecules.

## 4. Conclusions

In silico target fishing represents a profitable strategy for understanding the mode of action of bioactive compounds, assessing off-target effects, studying polypharmacology, and drug repurposing. In recent years, a large number of target fishing methods have been developed due to the availability of extensive libraries collecting information on the bioactivity of compounds, as well as to the advances achieved in computational techniques. By screening a compound against a protein database, it is possible to identify potential target candidates that match with this specific compound. In this review, we presented an overview on the main target fishing methods relying on ligand-based and receptor-based approaches, discussed their basic principles, and illustrated the main available web tools and their recent applications. The methodology used in a target fishing project depends on several factors, such as the type of target protein considered, the availability of 3D structures, and the number of reference bioactive ligands. Ligand-based methods have made incredible progress due to their flexibility, predictive performance, low computational requirements, and to the increased use of ML, which has proven to be an effective approach. However, each method has its advantages and limitations. As far as the ligand-based approaches are concerned, the problem of how to properly rank a series of potential targets predicted for the query ligand has not been totally explored. Indeed, similarity searching methods generally provide higher reliability in predicting potential targets if the matched ligands possess high similarity to the query molecule, but the identification of a suitable similarity threshold with the aim of reducing the number of false positives is still a challenge. An alternative is the use of ML in target fishing, if this implies the availability of a well-defined training space considering reference ligands confirmed to be inactive against the targets of interest by an experimental evaluation. Among the ML methods, PCM have shown good performance in the prediction of drug-target interactions, in combination with conventional approaches. More studies are needed to compare the reliability of various ranking criteria proposed in current research. Conversely, the main limitations of receptor-based methods are due to the availability of an only restricted pool of receptor 3D structures with respect to the known proteome, or to the inaccurate prediction of ligand-protein binding affinity, which again implies an improper ranking of the different potential targets predicted for the query ligand. Regarding receptor-based approaches, less applications employing ML methods are currently being reported. However, we believe that with the continuous advances in the field of artificial intelligence, ML, and deep learning algorithms will become a key element of receptor-based target fishing applications, as in other in silico approaches. Nevertheless, the limits of both ligand-based and receptor-based methods proved to be at least partially circumvented when the two different strategies were used in combination with each other, which may allow a more accurate description of the drug-target interaction, from both the drug and target point of view. Despite the availability of different types of approaches and resources that can be applied, target fishing still remains a challenge for drug discovery and repurposing. Therefore, a constant improvement in all aspects of target fishing strategies and, particularly, in the management of all levels of biological information, is required for achieving more efficient and reliable approaches.

## Figures and Tables

**Table 1:** Summary of the different ligand-based web tools herein discussed (access date: 1 June 2021).

Web Tool	Description	URL
SwissTargetPrediction	A combination of 2D and 3D similarity with known ligands	http://www.swisstargetprediction.ch
CSNAP3D	3D chemical similarity using a network algorithms score	http://services.mbi.ucla.edu/CSNAP
MolTarPred	2D Similarity search based on ECFP4 fingerprints	http://moltarpred.marseille.inserm.fr
TargetHunter	2D Similarity search based on ECFP6 fingerprints	http://www.cbligand.org/TargetHunter
TarPred	Molecular similarity searching with KNN-based fusion score	http://www.dddc.ac.cn/tarpred
STarFish	A stacking approach combining multiple multi-target QSAR models	https://github.com/ntcockroft/STarFish
MolTarPred	2D Similarity search based on ECFP4 fingerprints	http://moltarpred.marseille.inserm.fr

**Table 2:** Summary of the different receptor-based web tools further discussed in the review (access date: 1 June 2021).

Web Tool	Description	URL
INVDOCK	Reverse docking approach using an in-house database	http://bidd.group/group/softwares/invdock.htm
idTarget	Reverse docking approach based on Divide-and-conquer method and using all protein structures in the PDB	http://idtarget.rcas.sinica.edu.tw
TarFisDock	Reverse docking using the Potential Drug Target Database (PDTD)	http://www.dddc.ac.cn/tarfisdock
DPDR-CPI	Reverse docking using proteins from PDB and PDBBind, combined with ML models for target predictions	http://cpi.bio-x.cn/dpdr
ACID	Reverse docking with an automated consensus inverse docking protocol	http://chemyang.ccnu.edu.cn/ccb/server/ACID
DIA-DB	Identification of potential antidiabetic drugs based on similarity search and reverse docking	http://bio-hpc.eu/software/dia-db
GUT-DOCK	Identification of small-molecule interactions with gut hormone GPCRs based on reverse docking	https://gut-dock.miningmembrane.com
Drug ReposER	Pharmacophore approach based on sub-structural similarity to the binding interfaces of known drug binding sites.	http://mfrlab.org/drugreposer/
LigAdvisor	2D ligand-receptor interactions based on similarity estimations	https://ligadvisor.unimore.it
PLIP	Binding site alignment or similarity among molecular structures and residue sequences	https://projects.biotec.tu-dresden.de/plip-web
PharmMapper	Target identification based on pharmacophore mapping procedure	http://59.78.96.61/pharmmapper
